# Metabolomics Profiling Reveals Rehmanniae Radix Preparata Extract Protects against Glucocorticoid-Induced Osteoporosis Mainly via Intervening Steroid Hormone Biosynthesis

**DOI:** 10.3390/molecules24020253

**Published:** 2019-01-11

**Authors:** Tianshuang Xia, Xin Dong, Yiping Jiang, Liuyue Lin, Zhimin Dong, Yi Shen, Hailiang Xin, Qiaoyan Zhang, Luping Qin

**Affiliations:** 1 Department of Pharmacognosy, Second Military Medical University School of Pharmacy, Shanghai 200433, China; xiatianshuang@smmu.edu.cn (T.X.); dongxinsmmu@126.com (X.D.); msjyp@163.com (Y.J.); linliuyue2016@163.com (L.L.); dongzhiminedu@163.com (Z.D.); 18065148122@163.com (Y.S.); 2 Zhejiang Chinese Medical University School of Pharmacy, Hangzhou 310053, China

**Keywords:** Rehmanniae Radix Preparata, glucocorticoid-induced osteoporosis, metabolomics, osteoblast, steroid hormone biosynthesis

## Abstract

Rehmanniae Radix Preparata (RR), the dry rhizome of *Rehmannia glutinosa* Libosch., is a traditional herbal medicine for improving the liver and kidney function. Ample clinical and pharmacological experiments show that RR can prevent post-menopausal osteoporosis and senile osteoporosis. In the present study, in vivo and in vitro experiments, as well as a UHPLC-Q/TOF-MS-based metabolomics study, were used to explore the preventing effect of RR on glucocorticoid-induced osteoporosis (GIOP) and its underlying mechanisms. As a result, RR significantly enhanced bone mineral density (BMD), improved the micro-architecture of trabecular bone, and intervened in biochemical markers of bone metabolism in dexamethasone (DEX)-treated rats. For the in vitro experiment, RR increased the cell proliferation and alkaline phosphatase (ALP) activity, enhanced the extracellular matrix mineralization level, and improved the expression of runt-related transcription factor 2 (RUNX2) and osteopontin (OPN) in DEX-injured osteoblasts. For the metabolomics study, a total of 27 differential metabolites were detected in the DEX group vs. the control group, of which 10 were significantly reversed after RR treatment. These metabolites were majorly involved in steroid hormone biosynthesis, sex steroids regulation, and amino acid metabolism. By metabolic pathway and Western blotting analysis, it was further ascertained that RR protected against DEX-induced bone loss, mainly via interfering steroid hormone biosynthesis, as evidenced by the up-regulation of cytochrome P450 17A1 (CYP17A1) and aromatase (CYP19A1), and the down-regulation of 11β-hydroxysteroid dehydrogenase (HSD11B1). Collectively, these results indicated that RR had a notable preventing effect on GIOP, and the action mechanism might be related to steroid hormone biosynthesis.

## 1. Introduction

Glucocorticoids (GC) are widely used in clinics to treat rheumatoid arthritis, pulmonary, gastrointestinal, and autoimmune diseases for their excellent anti-inflammatory and immune-modulatory effects [1]. However, the life-time service of GC usually induces a series of complications, among which osteoporosis is the most devastating one. Currently, glucocorticoids-induced osteoporosis (GIOP) has become the third most-common etiology of pathological bone loss, only next to senile osteoporosis and postmenopausal osteoporosis. The underlying pathological mechanism of GIOP mainly lies in the direct inhibition of the osteoblastic cell cycle, differentiation, and function, and the stimulating action on endogenous gonadal steroids degeneration [2,3]. Clinical treatment for GIOP mainly includes calcium and active vitamin D supplementation and oral bisphosphonates, which is similar to that for postmenopausal osteoporosis and senile osteoporosis, and is incompatible with the pathological mechanism of GIOP. Besides, these therapeutic regimens may cause some potential adverse reactions, such as gastroesophageal irritation [4] and osteonecrosis of the jaw [5]. Thus, there is a desperate need to develop suitable therapeutic alternatives for GIOP with few adverse effects.

Rehmanniae Radix Preparata (RR), prepared from the dry rhizome of *Rehmannia glutinosa* Libosch., has traditionally been used for tonifying kidney essence in China [6]. This herb was first officially recorded in the Chinese Pharmacopoeia of 1963 version, and up to now, more than 140 compounds have been isolated and identified, including iridoid glycosides, phenylethanoid glycosides, monoterpenoids, and triterpenes. In traditional Chinese medicine (TCM), the kidney is in charge of bone, and bone loss is attributed to the kidney and liver deficiency. According to TCM theory, RR can promote the liver and kidney function. Hence, RR and TCM formulas containing RR are widely used to treat osteoporosis patients. In modern pharmacological studies, ample experiments have been used to understand the effect of RR on preventing osteoporosis. The water extract of RR was proved to improve BMD and increase the cortical bone thickness and trabeculation of the bone marrow spaces [7]. Liuwei Dihuang Pill, a traditional Chinese medicine formula mainly containing RR, was also proved to have remarkably preventive and therapeutic effects on primary osteoporosis through promoting bone formation [8,9]. In addition, it is noteworthy that RR can prevent the decrease of the splenic estrogen receptor and osteoblastic progestin receptor in aging female mice [10]. Inspired by these findings, we wonder if and how RR can alleviate bone loss induced by GC.

Metabolomics is a sensitive and unbiased analytical method that is used to comprehensively characterize the metabolite content of biological samples for understanding disease phenotypes. Metabolomics is characterized by “integrity and systematization”, which is consistent with the “multi-component and multi-target” theory in TCM [11]. Analysis of metabolite profiling before and after treatment with TCM can help explore their comprehensive therapeutic efficacies and action mechanisms. In this study, an untargeted metabolomics strategy based on UHPLC-Q-TOF/MS was employed to analyze the metabolic profile of GIOP rats, intending to better understand the action mechanism of RR on preventing DEX-induced bone loss and provide more promising candidates for the prevention and treatment for GIOP.

## 2. Results

### 2.1. UHPLC-MS Analysis of RR Extract

**Figure 1:**
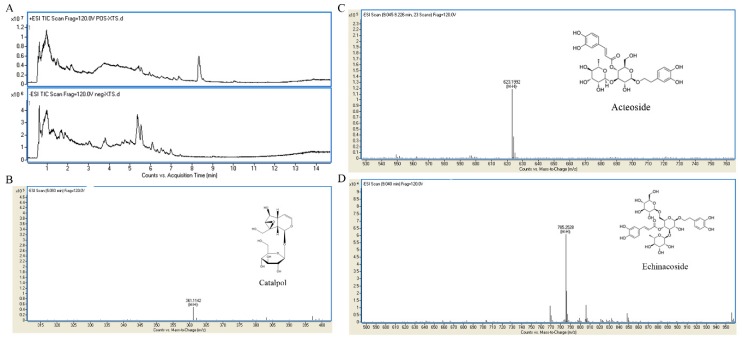
UHPLC-MS characteristics of RR extract. (**A**) The representative total ion chromatograms (TICs) in positive and negative ion mode; (**B**) catalpol in the RR extract sample; (**C**) acteoside in the RR extract sample; (**D**) echinacoside in the RR extract sample.

The UHPLC-MS characteristics of RR extract were detected. As shown in Figure 1, RR extract contains catalpol, acteoside, and echinacoside.

### 2.2. RR Improves the Micro-Architecture and BMD of the Femur in DEX-Treated Rats

**Figure 2:**
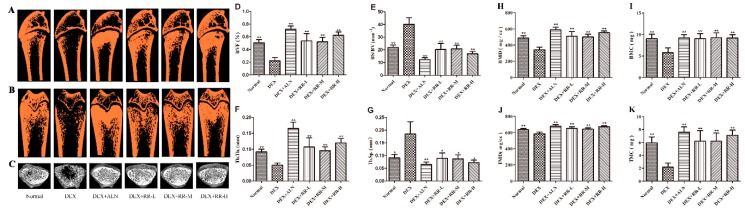
The region of interest (ROI) image and bone parameters analysis in the distal femur in DEX-treated rats. (**A**–**C**) Micro-CT images of ROI region in longitudinal section, transverse section, and 3-D architecture. (**D**–**K**) Trabecular bone parameters analysis of (**D**) BVF; (**E**) BS/BV; (**F**) Tb.Th.; (**G**) Tb.Sp.; (**H**) BMD; (**I**) BMC; (**J**) TMD; and (**K**) TMC in the distal femur region in DEX-treated rats. Values were expressed as the mean ± SD; *n* = 7. * p < 0.05, ** p < 0.01 compared with DEX group.

As shown in Figure 2A–C, the micro-CT analysis showed a severe impairment of bone micro-architecture in the femur of DEX-treated rats, and the trabecular region exhibited a small, thin, and sparse morphology. RR could obviously improve the trabecular micro-architecture, with a slightly disordered trabecular arrangement and mild expanding medullary cavity. As shown in Figure 2D–G, the morphologic parameters of trabecular thickness (Tb.Th.) and bone volume fraction (BVF) decreased significantly, and trabecular separation (Tb.Sp.) and bone surface to bone volume (BS/BV) increased significantly in the femur of DEX-treated rats compared with those in the normal group (*p* < 0.01). RR treatment could significantly reverse the alterations of trabecular morphologic parameters, increase Tb.Th. and BVF, and decrease Tb.Sp. and BS/BV (*p* < 0.01). In addition, as shown in Figure 2K–H, DEX significantly reduced the BMD, bone mineral content (BMC), tissue mineral density (TMD), and tissue mineral content (TMC) compared with those in the normal group (*p* < 0.01), while RR administration significantly enhanced these indices (*p* < 0.01).

### 2.3. RR Regulates Bone Metabolism-Related Biochemical Markers in DEX-Treated Rats

**Figure 3:**
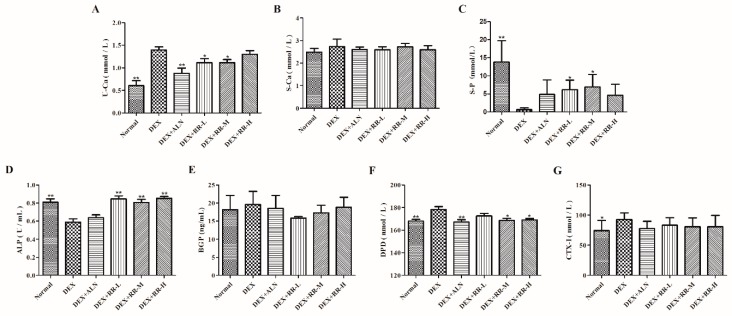
Effects of RR on biochemical markers levels of bone metabolism in DEX-treated rats. (**A**) U-Ca; (**B**) S-Ca; (**C**) S-P; (**D**) ALP; (**E**) BGP; (**F**) DPD; (**G**) CTX-I. Values were expressed as the mean ± SD; *n* = 7. * p < 0.05, ** p < 0.01 compared with DEX group.

As shown in Figure 3A–C, DEX significantly increased the urine calcium (U-Ca) level and decreased the serum phosphorus (S-P) level compared with those in the normal group (*p* < 0.01). RR at low- and medium-doses significantly reduced the U-Ca level, and increased the S-P level, in DEX-treated rats (*p* < 0.05). There was no significant difference in the serum calcium (S-Ca) level between the six groups. Alkaline phosphatase (ALP), bone gla-protein (BGP), deoxypyridinoline (DPD), and c-terminal telopeptides of type I collagen (CTX-I) are biochemical markers of bone turnover. As shown in Figure 3D–G, DEX significantly decreased the serum ALP level, and increased the urine DPD and serum CTX-I levels, compared with those in the normal group (*p* < 0.01 or *p* < 0.05). RR at medium- and high-dosages could significantly increase the ALP level and decrease the DPD level in DEX-treated rats (*p* < 0.01 or *p* < 0.05). RR also insignificantly decreased BGP and CTX-I levels in DEX-treated rats.

### 2.4. RR Enhances the Proliferation, Differentiation, and Mineralization Levels of Osteoblasts Injured by DEX

**Figure 4:**
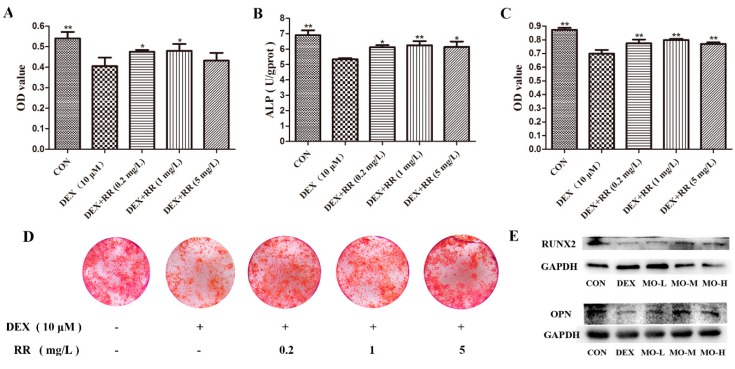
Effects of RR on the proliferation, differentiation, and mineralization levels of DEX-injured osteoblasts. (**A**) MTT assay; (**B**) ALP activity; (**C**) extracellular matrix mineralization; (**D**) representative images of osteoblastic bone mineralization nodule stained with alizarin red; (**E**) the protein expressions of RUNX2 and OPN. Values were expressed as the mean ± SD; (**A**) *n* = 4, (**B**–**F**) *n* = 3. * p < 0.05, ** p < 0.01 compared with DEX group.

To further validate the in vitro effect of RR on preventing bone loss, the activities of DEX-injured osteoblasts were assayed. The results showed that DEX significantly decreased the proliferation (Figure 4A), ALP activity (Figure 4B), and mineralization level (Figure 4C,D) of osteoblasts (*p* < 0.01). After treatment, RR at doses of 0.2 mg/L and 1 mg/L significantly increased the cells’ proliferation compared with that of the DEX-treated control (*p* < 0.05). RR at all dosages prominently increased the ALP activity (*p* < 0.01 or *p* < 0.05), and the intensity and area of staining, as well as promoted the formation of mineralized nodules in DEX-treated osteoblasts (*p* < 0.01). In addition, DEX inhibited the expression of RUNX2 and OPN compared with that in the control group, while RR treatment improved the RUNX2 and OPN expression in DEX-injured osteoblasts to some extent (Figure 4E).

### 2.5. Metabolomics Analysis

**Figure 5:**
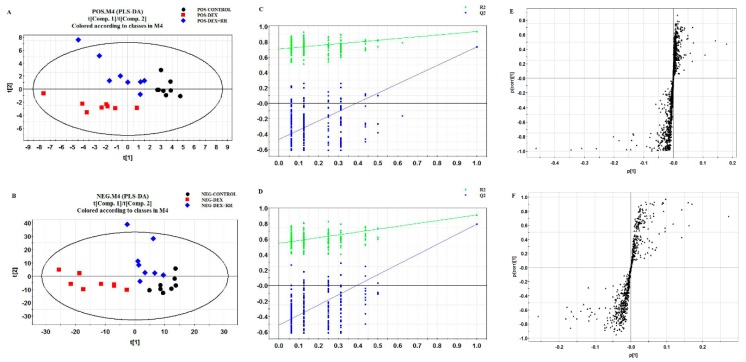
Multivariate analysis based on the UHPLC-Q/TOF-MS profiling data for samples in the control, DEX, and DEX+RR groups in positive and negative ion mode (*n* = 8). (**A**) PLS-DA score plot in positive ion mode; (**B**) validation of PLS-DA model in positive ion mode; (**C**) S-plot of PLS-DA model in positive ion mode; (**D**) PLS-DA score plot in negative ion mode; (**E**) validation of PLS-DA model in negative ion mode; (**F**) S-plot of PLS-DA model in negative ion mode.

The TICs in both positive and negative ion modes of urine samples from three experimental groups are shown in Figure S1. The QC superposed graph is shown in Figure S2. To determine whether metabolite fingerprints in urine differed between the control, DEX, and DEX+RR-M groups in the metabolomics approach, the partial least squares discriminant analysis (PLS-DA) model was applied. The metabolic profiles showed that urine samples of the DEX group evidently separated from those of the normal group. Samples of the DEX+MO group were situated close to those of the control group and far away from those of the DEX group (Figure 5A,B). Validation of the PLS-DA model exhibited a good fitting degree (Figure 5C,D).

### 2.6. Identification of Potential Biomarkers in DEX-treated Rats

Variables far from the origin in the S-plot (Figure 5E,F) with a variable importance plot (VIP) > 1 and *p* < 0.05 were applied to select potential biomarkers. As a result, 27 differential metabolites between rats in the control group and DEX group were selected as potential biomarkers, 23 of which were the only ones for which the endogenous compound matched with the accurate mass via online database Metlin and Human Metabolome Database (HMDB), except for dipeptide. These differential metabolites were mainly involved in amino acid metabolism, fatty acid biosynthesis, steroid hormone biosynthesis, and arachidonic acid metabolism (Table 1).

### 2.7. RR Reverses Metabolic Dysregulation in DEX-Treated Rats

**Figure 6:**
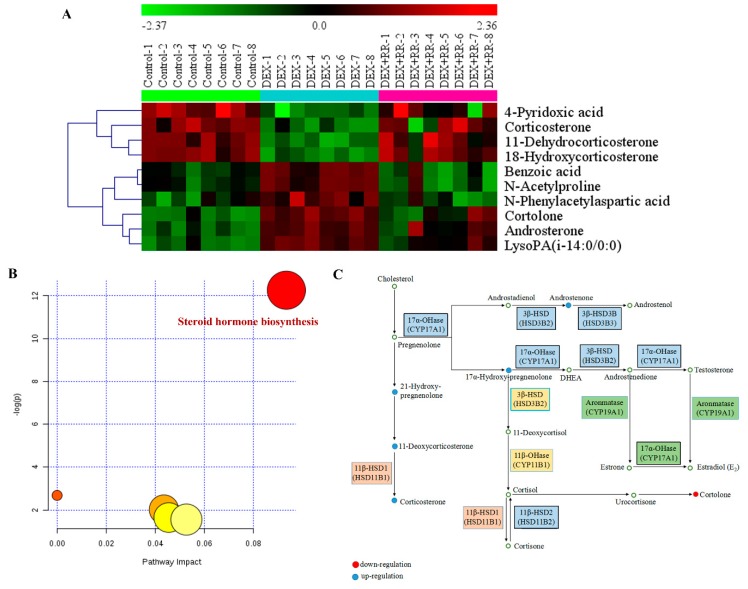
Heat map and metabolic pathway analysis based on the UHPLC-Q/TOF-MS profiling data for samples in the control, DEX, and DEX+RR groups. (**A**) Hierarchical clustering heat map of the differential metabolites before and after RR treatment in DEX-treated rats; (**B**) summary of ingenuity pathway analysis with MetPA. The size and color of each circle were based on pathway impact value and *p*-value, respectively; (**C**) construction of the metabolic pathway related to differential metabolites. The metabolites were labeled in blue (up-regulation after RR treatment) or red (down-regulation after RR treatment).

The metabolic changes after treatment with RR at 4 g/kg in GIOP rats were studied. Seen in Table 1, the levels of benzoic acid, N-acetylproline, 4-pyridoxic acid, androsterone, N-phenylacetylaspartic acid, 11-dehydrocorticosterone, 18-hydroxycorticosterone, cortolone, corticosterone, and lysoPA were significantly reversed after RR treatment, and these metabolites were majorly involved in steroid hormone biosynthesis, sex steroids regulation, and amino acid metabolism. The heat map was constructed based on the normalized data set of the reversed metabolites, and the results showed that the variation tendency of most metabolites after RR treatment was different from that in DEX group, while the same as that in the control group (Figure 6A). The ingenuity metabolic pathway analysis showed that the potential biomarkers were majorly involved in steroid hormone biosynthesis (Figure 6B). The metabolic pathway map associated with differential metabolites was depicted based on the Kyoto Encyclopedia of Genes and Genomes (KEGG) (Figure 6C). These results showed that RR reversed metabolic dysregulation in DEX-treated rats, mainly via intervening steroid hormone biosynthesis.

### 2.8. RR Regulates the Expressions of Key Proteins in Steroid Hormone Biosynthesis

**Figure 7:**
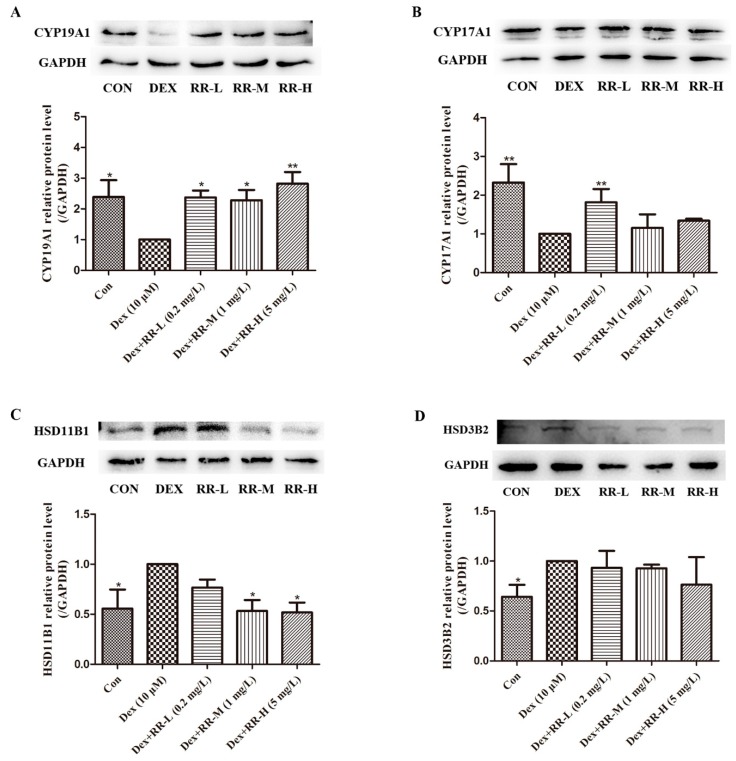
Effects of RR on the expression of key proteins related to steroid hormone biosynthesis. Protein expressions and relative levels of (**A**) CYP19A1; (**B**) CYP17A1; (**C**) HSD11B1; (**D**) HSD3B2 were analyzed by Western blotting. All values are expressed as the mean ± SD; *n* = 3. * p < 0.05, ** p < 0.01 compared with DEX group.

To further validate the regulatory effect of RR on steroid hormone biosynthesis, the expression of key proteins related to steroid hormone biosynthesis was analyzed by Western blotting. As shown in Figure 7, DEX significantly disturbed the expression of CYP17A1, CYP19A1, HSD11B1, and HSD3B2 (*p* < 0.01 or *p* < 0.05). After treatment, RR at all doses prominently increased the expression of CYP19A1 compared with that in the DEX group (*p* < 0.01 or *p* < 0.05). RR at a dose of 0.2 mg/L prominently increased the expression of CYP17A1 (*p* < 0.01). RR at doses of 1 mg/L and 5 mg/L also significantly inhibited the HSD11B1 expression (*p* < 0.05). However, there was no effect of RR on HSD3B2 expression.

## 3. Discussion

GIOP, recognized as the most common iatrogenic cause of secondary osteoporosis, has imposed a serious threat to public health. The present investigation found that RR could prevent GIOP in both DEX-treated rats and DEX-injured osteoblasts. Our metabolomics analysis showed that DEX caused great metabolic disorders, while RR could rebalance the metabolic disruptions, mainly via regulating steroid hormone biosynthesis and amino acid metabolism. Through further pathway and Western blotting analysis, it was ascertained that RR preventing GIOP was related to intervening steroid hormone biosynthesis.

Both human and animal studies demonstrated deleterious skeletal effects within weeks of pharmacological GC administration, as evidenced by the alteration of BMD and the bone micro-architecture. The histomorphometric parameters of the trabecular bone could predict GC-induced osteopenia and the deterioration of bone quality. The present study found that RR could improve the micro-architecture, enhance the BMD, and increase the trabecular parameters in the femur in DEX-treated rats, suggesting that administration with RR was effective in both preserving bone mass and rescuing the deterioration of bone micro-architecture. It is generally assumed that bone loss in the chronic state of GIOP is mostly attributable to the decrease of osteoblastic bone formation, and excessive GC can inhibit the osteoblastic proliferation, differentiation, and bone matrix mineralization [12]. In this study, RR was able to improve the osteoblastic proliferation, ALP activity, and bone matrix mineralization level, which further prove that RR has a potent anti-GIOP effect. RUNX2 is a regulator in osteoblast differentiation at an early stage, and plays a crucial role in skeletal morphogenesis, tooth development, chondrogenesis, and vasculogenesis [13]. It has been proved that RUNX2 can upregulate the expression of the PI3K-Akt pathway, and enhance its DNA binding ability in immature mesenchymal stem cells (MSCs) and immature osteoblasts [14]. The increased expression of bone matrix proteins, such as ALP, BGP, and OPN, also stimulates mineralization and leads to bone formation [15]. It was found in our study that RR could increase the ALP level in the serum of DEX-treated rats, and enhance the expression of RUNX2 and OPN in DEX-injured osteoblasts, further suggesting that RR was able to promote osteoblastic differentiation.

To delineate the mechanisms underlying the preventive effect of RR against GIOP, a metabolomics profiling of rat urine based on UHPLC-Q-TOF/MS analysis was applied. It is well-known that the deficiency of androgens or estrogens will induce bone loss [16]. Epiandrosterone is a metabolite of the most abundant adrenal androgenic steroid dehydroepiandrosterone (DHEA) [17], and androgens and estrogens are both made from DHEA [18]. It was found in our study that DEX decreased the epiandrosterone or androsterone level in GIOP rats, while RR could reverse its abnormal descent after eight-week treatment. It indicated that DEX might cause androgens and estrogens deficiency via suppressing DHEA secretion, whereas RR made a significant callback of these hormone levels, and further protected against glucocorticoid-induced bone loss. Pregnenolone is a well-known precursor for the biosynthesis of various sex hormones, such as estrogen, progesterone, and testosterone [19]. It has been proved that pregnenolone and its heterocyclic analogues have the potential to become novel anti-osteoporotic agents [20]. Hydroxypregnenolone is converted from pregnenolone by cytochrome P450, and is also involved in the biosynthesis of gonadal steroid hormones and adrenal corticosteroids [18,21]. In our study, a significant decrease of hydroxypregnenolone was found in GIOP rats, and compared with that, there was a rising tendency after RR treatment, indicating that RR could improve the pregnenolone level, and further sustain bone homeostasis.

Dehydrocorticosterone and corticosterone both belong to adrenocortical steroids. It was reported that corticosterone and 11-dehydrocorticosterone levels were decreased in GIOP model rats [22], which was consistent with the finding in the present study. Contrarily, elevated corticosterone, 18-hydroxycorticosterone, and 11-dehydrocorticosterone levels were detected after RR treatment. In addition, it was found in our study that the correlation coefficient of 18-hydroxycorticosterone and 11-dehydrocorticosterone vs. ALP and DPD levels was more than 0.6, suggesting that these two metabolites might be related to bone metabolism. The conversion of inactive 11-dehydrocorticosterone into active corticosterone is catalyzed by 11β-hydroxysteroid dehydrogenase (HSD11B1), which was confirmed to play a crucial role in metabolically relevant tissues, such as skeletal muscles [23]. Previous studies discovered a close relationship between HSD11B1 activity and osteoblast differentiation after continuous injury of human osteoblasts with DEX, and proved that DEX could induce an overexpression of HSD11B1 and decrease osteoblast differentiation [24]. In the present study, an elevated HSD11B1 level was found in DEX-injured osteoblasts, while RR treatment could depress its overexpression, further indicating that RR preventing bone loss might be related to regulating HSD11B1 activity and its conversion of inactive 11-dehydrocorticosterone into active corticosterone.

Except for HSD11B1, some other key proteins participating in steroid hormone biosynthesis also influence bone metabolism. Aromatase, known as cytochrome P450 19A1 (CYP19A1), is closely related to postmenopausal osteoporosis. The aromatase deficiency can result in estrogen deficiency [25], and the estrogen deficiency at menopause accelerated the age-dependent involution of the female skeleton and contributed to the loss of bone mass, architectural integrity, and strength [26]. CYP17A1 encodes an enzyme with both 17α-hydroxylase and 17,20-lyase activities, and 17α-hydroxylase is responsible for hydroxylating pregnelone and progesterone, which are then converted to C19 steroid precursors of testosterone and estrogen by 17,20-lyase activity [27]. The deficiency of CYP17A1 can result in reduced growth and osteoporosis. It was found in our study that RR could improve the CYP19A1 and CYP17A1 levels to some extent, further suggesting that RR preventing GIOP was related to intervening steroid hormone biosynthesis.

In conclusion, pharmacological experiments and UHPLC-Q/TOF-MS-based metabolomics analysis were used to evaluate the effects and underlying mechanisms of RR on protecting against GIOP. In GIOP model rats, RR was able to improve the cancellous bone structure, enhance BMD, and ameliorate bone metabolism homeostasis. In DEX-injured osteoblasts, RR could improve the cell proliferation, differentiation, and mineralization level, and increase the expression of RUNX2 and OPN. Metabolomics profiling indicated that RR might prevent DEX-induced bone loss through regulating sex steroids regulation, steroid hormone biosynthesis and amino acid metabolism. Metabolic pathway and Western blotting analysis further clarified that RR protected against GIOP, mainly via intervening steroid hormone biosynthesis. The above results demonstrated, for the first time, that RR helped protect against GIOP, and provided an excellent candidate for GIOP therapeutics.

## 4. Methods and Materials

### 4.1. Chemicals and Reagents

Chemicals and reagents used in this study included chromatographic-grade methanol and acetonitrile (Merck, Darmstadt, Germany); standard chemicals of catalpol, echinacoside, and acteoside (HPLC ≥98%, Shanghai Yuan-ye Bio-Technology Co., Ltd., Shanghai, China); and DEX (Dalian Meilun Biotech Co., Ltd., Dalian, China); ALN (MSD Pharmaceutical Co., Ltd., Hangzhou, China); Ca, P, ALP, and TRAP assay kits (Nanjing Jian Cheng Bioengineering Institute, Nanjing, China); enzyme-linked immunosorbent assay (ELISA) kits for BGP, DPD, and CTX-I (Xin Yu Biological Engineering Co., Ltd., Shanghai, China); antibodies specific for OPN, CYP17A1, CYP19A1, HSD11B1, and HSD3B2 (Abcom, Cambridge, UK); antibodies specific for RUNX2, anti-rabbit IgG, anti-mouse IgG, and GAPDH (CST, Danvers, MA, USA).

### 4.2. Preparation of RR Extract

RR is the processed product of the dry rhizome of *Rehmannia glutinosa* Libosch. In this study, RR was purchased from Shanghai De Kang Pharmaceutical Co., Ltd. and authorized by Professor Qin Luping, Department of Pharmacognosy, School of Pharmacy of the Second Military Medical University. The voucher specimen (RR 20170328) was deposited in the herbarium of Second Military Medical University. Dried RR (350 g) was cut into small pieces and reflux-extracted with 2.8 L distilled water at 60 °C three times. The extract of RR was concentrated with a vacuum evaporator, and dissolved in an appropriate volume of distilled water, and then adjusted to a concentration of 0.1 g/mL, 0.2 g/mL, and 0.4 g/mL, equivalent to crude drugs for animal experiments, or 0.2 mg/L, 1 mg/L, and 5 mg/L, equivalent to crude drugs for in vitro experiments. The dose of the RR extract was converted according to the clinical dosage of humans.

### 4.3. UHPLC-MS Analysis of RR Extract

UHPLC-MS analysis was performed on an Agilent 1290 Infinity LC system coupled to an Agilent 6538 accurate-mass quadrupole time-of-flight (Q-TOF) mass spectrometer (Agilent, Palo Alto, CA, USA). Chromatographic separations were performed on an Acquity UHPLC HSS T3 column (2.1 × 100 mm, 2.5 μm, Waters, Milford, MA, USA) at 25 °C, and the injection volume was 3 μL at a flow rate of 0.4 mL/min. The mobile phase consisted of 0.1% formic acid (A) and ACN modified with 0.1% formic acid (B). The gradient program was as follows: 5% B over 0–2 min, 5–95% B over 2–13 min, and 95% B over 13–19 min. The capillary voltage was 4 kV for positive ion mode and 3.5 kV for negative ion mode. The drying gas flow rate was 11 L/min at 350 °C. The nebulizer pressure was 45 psig, the fragmentor voltage was 120 V, and the skimmer voltage was 60 V. Data were collected in a centroid mode and the mass range was *m*/*z* 100–1100 using an extended dynamic range.

### 4.4. Animal Treatment and Samples Collection

Female Sprague-Dawley (SD) rats aged 12 weeks and weighing 180–220 g were purchased from Slacom (Shanghai, China), and maintained at the Experimental Animal Center of the Second Military Medical University. The animals were housed at 24 ± 0.5 °C with a 12-h light and 12-h dark cycle with free access to food and water. After seven-day acclimatization, animals were randomly divided into six groups according to their body weights (*n* = 8 per group): Normal (saline) group, DEX (DEX 2.5 mg/kg + saline) group, DEX + ALN (DEX 2.5 mg/kg + ALN 1 mg/kg) group, DEX + RR-L (DEX 2.5 mg/kg + RR 1 g/kg) group, DEX + RR-M (DEX 2.5 mg/kg + RR 2 g/kg) group, and DEX + RR-H (DEX 2.5 mg/kg + RR 4 g/kg) group. At the first week, all rats except for those in the normal group were injected with DEX daily via the caudal vein. Then, the rats were injected with DEX via the caudal vein twice a week and orally administered with different dosages of RR daily for eight weeks.

The day before the end of the experiment, rats were housed individually in metabolic cages for 24 h without providing food to collect urine samples. Then, all rats were anesthetized with an intraperitoneal (i.p.) injection of 300 mg/kg chloral hydrate. The blood was collected from the abdomen artery, and centrifuged at 3000 rpm for 10 min to collect the sera. Serum samples were stored at −80°C for biochemical determination and metabolomics profiling. The right femur was prepared for micro-computed tomography scanning. All studies were conducted in accordance with the NIH publication and Second Military Medical University principles for laboratory animal use and care.

### 4.5. Micro-CT Analysis and Biochemical Marker Measurement

The measurement of trabecular micro-architecture was performed on an micro-CT system (eXplore Locus, GE Healthcare, Boston, MA, USA) with the voltage set at 80 kV, current at 80 μA, angular rotation at 360°, and angular step at 0.4°. The femur was scanned from the proximal growth plate in the distal direction (14 μm/slice). The ROI was selected at a distance of 0.16 mm from the distal end of the growth plate. BMD, BMC, TMD, TMC, Tb.Th., Tb.Sp., BVF, and BS/BV were measured.

The U-Ca, S-Ca, S-P, ALP, and TRAP levels were measured by standard colorimetric methods using commercially available assay kits. The BGP, DPD, and CTX-I levels were measured using the ELISA kits and the micro-plate reader (Thermo Fisher Scientific Inc., Pittsburgh, PA, USA).

### 4.6. UHPLC-Q-TOF/MS Metabolomics Analysis

Rat urine samples (100 μL) in the control, DEX, and DEX+RR-M groups were added into 300 μL methanol solutions containing 5 μg/mL 2-chloro-L-phenylalanine as the internal standard. The mixed solution was homogenized by vortex for 5 min and centrifuged at 13,000 rpm, 4 °C for 15 min. A total of 150 μL supernatant was filtered through a 0.22 μm membrane for metabolomics studies. The method of UHPLC-Q-TOF/MS metabolomics analysis was the same as that mentioned in Section 4.3. The mobile phase consisted of 0.1% formic acid (A) and ACN modified with 0.1% formic acid (B), and the gradient program was as follows: 5% B over 0–2 min, 5–15% B over 2–8 min, 15–30% B over 8–10 min, 30–95% B over 10–13 min, and 95% B over 13–15 min. The reference ions were 121.0509 and 922.0098 in positive mode, and 112.9856 and 1033.9881 in negative mode. The QC samples were used every eight urine samples to evaluate the stability during sequence analysis.

### 4.7. Data Processing and Multivariate Analysis

All UHPLC-MS raw data were transformed into common data format (.mzdata) files using Agilent MassHunter qualitative software. The program XCMS (http://metlin.scripps.edu/download/) was used for peak alignment, peak extraction, and automatic integration. After mean-centering and pareto-scaling procedures, the retention time (RT)-*m*/*z* pairs, observations, and relative ion intensities of all detected ions were imported into SIMCA-P 11.0 software package (Umetrics, Umea, Sweden) for principal component analysis (PCA) and PLS-DA. HMDB (http://www.hmdb.ca/) and Metlin database (https://metlin.scripps.edu/) were selected for metabolites matching based on a combination of database queries using exact mass measurements. Additionally, the model of PLS-DA was evaluated according to the cross-validation of R^2^, Q^2^ value, and permutation test. An independent sample *t*-test was performed for statistical analysis using SPSS version 20.0 (IBM, USA) and *p* < 0.05 was considered statistically significant. A heat map of the different metabolites was processed by MEV-MultiExperiment Viewer 4.8.1. The pathway analysis of potential biomarkers was performed with MetaboAnalyst (http://www.metaboanalyst.ca/) and KEGG pathway database (http://www.genome.jp/kegg/).

### 4.8. Osteoblasts Culture

Female Wistar rats aged 24 h were purchased from the Experimental Animal Center of the Second Military Medical University (Shanghai, China). Primary osteoblasts were prepared from the calvarias according to literature [28], and cultured in α-MEM supplemented with 10% FBS at 37 °C in a humidified atmosphere containing 5% CO_2_. All studies were conducted in accordance with the NIH publication and Second Military Medical University principles for laboratory animal use and care.

### 4.9. Osteoblastic MTT and ALP Activity Assay

Osteoblasts were injured with DEX (10 μM), and at the same time treated with RR (0.2, 1 and 5 mg/L) for 48 h in an MTT assay and for seven days in an ALP activity assay. In the MTT assay, 20 μL of 5 mg/mL MTT was added into plates after drugs treatment. The medium was discarded and the formazan crystals that formed in the cells were dissolved in 150 μL of DMSO. Optical density (OD) was measured at 570 nm. For the ALP activity assay, the cells were lysed after drug treatment, and the total protein concentration was measured using a BCA-protein assay kit. The ALP activity was measured according to the conversion of colorless *p*-nitrophenyl phosphate to colored *p*-nitrophenol.

### 4.10. Osteoblastic Bone Matrix Mineralization Assay

Osteoblasts were cultured in osteogenic differentiation medium (α-MEM with 10% FBS, 50 μg/mL ascorbic acid, and 10 mM β-glycerophosphate) for 12 days. Cells were injured with DEX (10 μM), and at the same time treated with RR (0.2, 1 and 5 mg/L) for another nine days in osteogenic differentiation medium. After washing, cells were fixed with ice-cold 4% paraformaldehyde for 10 min. Then, 0.1% Alizarin Red staining solution (pH 8.3) was added and incubated for 30 min at 37 °C. The staining of Alizarin Red was incubated with 5% (*v/w*) cetylpyridinium chloride for 30 min at 37 °C and OD was measured at 570 nm.

### 4.11. Western Blotting

Osteoblasts were injured with DEX (10 μM), and at the same time treated with RR (0.2, 1 and 5 mg/L) for 48 h. Then, cells were lysed and centrifuged at 12,000 rpm, 4 °C for 10 min. The protein concentration of the supernatants was determined using the BCA kit. Each sample (20 μg protein) was separated by SDS-PAGE (10% gel) and transferred to polyvinylidene fluoride (PVDF) membranes. The membranes were then blocked in 5% BSA, and incubated with the primary antibodies overnight at 4 °C. After washing with TBS-T, the membranes were incubated with a second antibody for 1 h at room temperature. Immunoreaction signals were detected using electrochemiluminescence (ECL) reagent (Tanon, Shanghai, China), and exposed on a Gel imaging system (Tanon-5200 Multi, Shanghai, China).

### 4.12. Statistical Analysis

The data were expressed as the mean ± standard deviation (SD) and group differences were determined by one way analysis of variance (ANOVA) with the Turkey test. The analyses were performed using SPSS version 20.0 and *p* < 0.05 was considered statistically significant.

## Figures and Tables

**Table 1:** Screening of potential biomarkers in rat urine.

NO.	Metabolite Identification	Rt (min)	*m/z*	Adduct	VIP	Fold Change Value		*P*-Value		Related Pathway	HMDB/ MELIN ID
						DEX/Control	DEX+RR/DEX	DEX/Control	DEX+RR/DEX		
1	Glycine	3.818	76.0395	[M+H]^+^	1.78	0.491	1.280	0.025 *	0.845	Amino acid metabolism	HMDB0000123
**2**	**Benzoic acid**	2.449	140.0701	[M+NH_4_]^+^	1.23	2.219	**0.496**	0.006 **	0.013 *	Amino acid metabolism	HMDB0001870
**3**	**N-Acetylproline**	2.450	158.0807	[M+H]^+^	2.29	2.155	**0.492**	0.008 **	0.012 *	Amino acid metabolism	HMDB0094701
4	Naphthol	0.842	162.0926	[M+NH_4_]^+^	2.20	2.192	0.574	0.047 *	0.158	Metabolism of xenobiotics by cytochrome P450	HMDB0012138/ HMDB0012322
5	Indoleacetic acid	3.823	198.0513	[M+Na]^+^	1.30	0.467	1.280	0.013 *	0.848	Amino acid metabolism	HMDB0000197
**6**	**4-Pyridoxic acid**	1.156	201.0860	[M+H]^+^	1.05	0.373	**2.167**	0.000 **	0.014 *	Vitamin B6 metabolism	HMDB0000017
7	N-Hydroxy-L-tyrosine	1.563	215.1006	[M+NH_4_]^+^	2.08	0.307	1.869	0.000 **	0.312	Amino acid metabolism	HMDB0038750
8	N-alpha-Acetylcitrulline	1.154	218.1137	[M+H]^+^	1.21	0.396	1.964	0.001 **	0.057	Amino acid metabolism	HMDB0000856
9	3-Oxododecanoic acid	6.690	232.1898	[M+NH_4_]^+^	1.24	2.714	0.720	0.010 *	0.442	Fatty acid biosynthesis	HMDB0010727
10	Valerylcarnitine/Isovalerylcarnitine	5.306	246.1698	[M+H]^+^	1.15	2.136	0.725	0.042 *	0.479	Fatty acid biosynthesis	HMDB0013128/HMDB0000688
**11**	**N-Phenylacetylaspartic acid**	4.080	252.0842	[M+H]^+^	1.53	2.064	**0.551**	0.007 **	0.022 *	Amino acid metabolism	HMDB0029355
12	Palmitic acid/Isopalmitic acid	9.939	274.2743	[M+NH_4_]^+^	5.52	0.741	1.156	0.013 *	0.463	Fatty acid biosynthesis	HMDB0000220/HMDB0031068
**13**	**Androsterone/Epiandrosterone**	8.665	313.2156	[M+Na]^+^	1.61	3.719	**0.592**	0.001 **	0.048 *	**Steroid hormone biosynthesis**	HMDB0000031/ HMDB0000365
14	Hydroxypregnenolone	10.545	333.2407	[M+H]^+^	1.01	0.414	1.438	0.029 *	0.795	Steroid hormone biosynthesis	—
15	Galactosylhydroxylysine	6.575	342.1904	[M+NH_4_]^+^	1.01	6.878	0.867	0.006 **	0.942	Energy metabolism	HMDB0000600
**16**	**11-Dehydrocorticosterone**	9.011	345.2061	[M+H]^+^	1.90	0.325	**3.389**	0.003 **	0.001 **	**Steroid hormone biosynthesis**	HMDB0004029
**17**	**Corticosterone**	9.537	347.2219	[M+H]^+^	2.53	0.289	**4.163**	0.128	0.035 *	**Steroid hormone biosynthesis**	HMDB0001547
18	Arachidonic acid/Arachidonate	10.055	349.2378	[M+FA-H]^-^	1.87	0.306	0.853	0.014 *	0.989	Arachidonic acid metabolism	HMDB0001043/HMDB0060102
19	Docosapentaenoic acid	9.795	353.2475	[M+Na]^+^	3.45	3.137	0.413	0.032 *	0.077	Fatty acid biosynthesis	HMDB0006528/ HMDB0001976
20	MG(0:0/16:0/0:0)	13.949	353.2667	[M+Na]^+^	2.57	0.670	0.964	0.010 *	0.991	Fatty acid biosynthesis	—
**21**	**18-Hydroxycorticosterone**	9.008	361.2016	[M-H]^-^	1.59	0.215	**4.840**	0.001 **	0.000 **	Steroid hormone biosynthesis	HMDB0000319
22	HETE	8.666	365.2332	[M+FA-H]^-^	3.04	3.978	0.503	0.000 **	0.110	Arachidonic acid metabolism	—
**23**	**Cortolone**	8.667	367.2478	[M+H]^+^	1.40	4.327	**0.553**	0.000 **	0.042 *	**Steroid hormone biosynthesis**	HMDB0003128
24	Carbocyclic Thromboxane A2	9.793	371.2576	[M+Na]^+^	4.34	2.766	0.414	0.048 *	0.078	Arachidonic acid metabolism	METLIN-45632
25	α,α-Dimethyl anandamide	13.286	376.3186	[M+Na]^+^	1.34	1.694	1.017	0.026 *	0.999	Retrograde endocannabinoid signaling	METLIN-36748
**26**	**LysoPA(i-14:0/0:0)**	8.665	405.2038	[M+Na]^+^	2.41	7.529	**0.452**	0.000 **	0.027 *	Lysophosphatidic metabolism	HMDB0114765
27	O-Arachidonoyl Glycidol	9.789	405.2644	[M+FA-H]^-^	3.23	3.122	0.286	0.035 *	0.120	Retrograde endocannabinoid signaling	METLIN-44872

* *p* < 0.05, ** *p* < 0.01 compared with DEX group.

## Supplementary Materials

The following are available online, Figure S1: Representative total ion chromatograms (TIC) in metabolomics study; Figure S2: Fig. S2 Quality control (QC) superposed graph in metabolomics study.
