# Supplementary figures and images for: Metabolomics Profiling Reveals Rehmanniae Radix Preparata Extract Protects against Glucocorticoid-Induced Osteoporosis Mainly via Intervening Steroid Hormone Biosynthesis
Source: Molecules. 2019 Jan 11;24(2):253. doi: 10.3390/molecules24020253 (PMC6358733; doi:10.3390/molecules24020253)

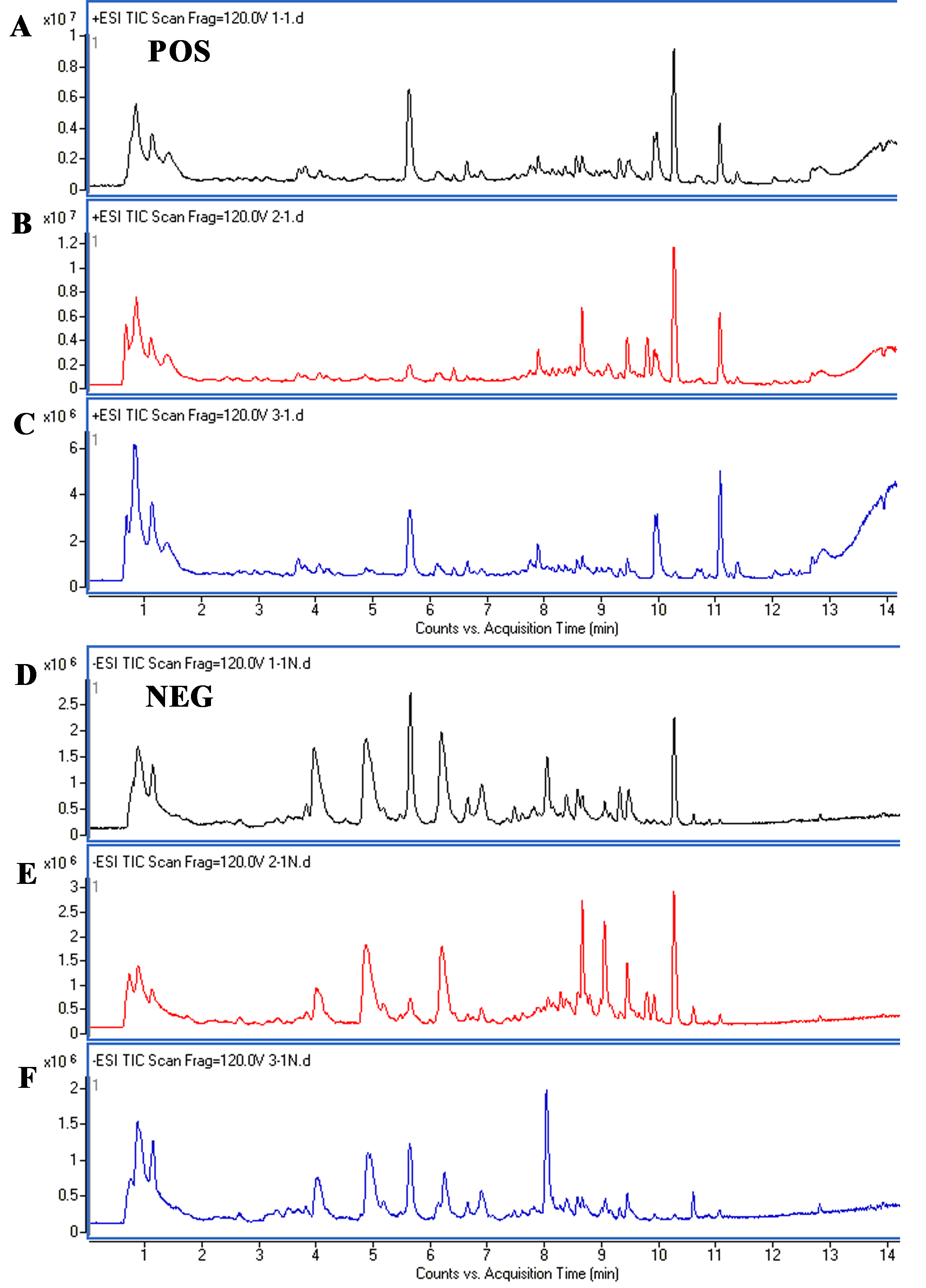

Supplement: Supplementary file 1 [file molecules-24-00253-s001.zip › Supplementary Files/Fig. S1 Representative total ion chromatograms.tif]

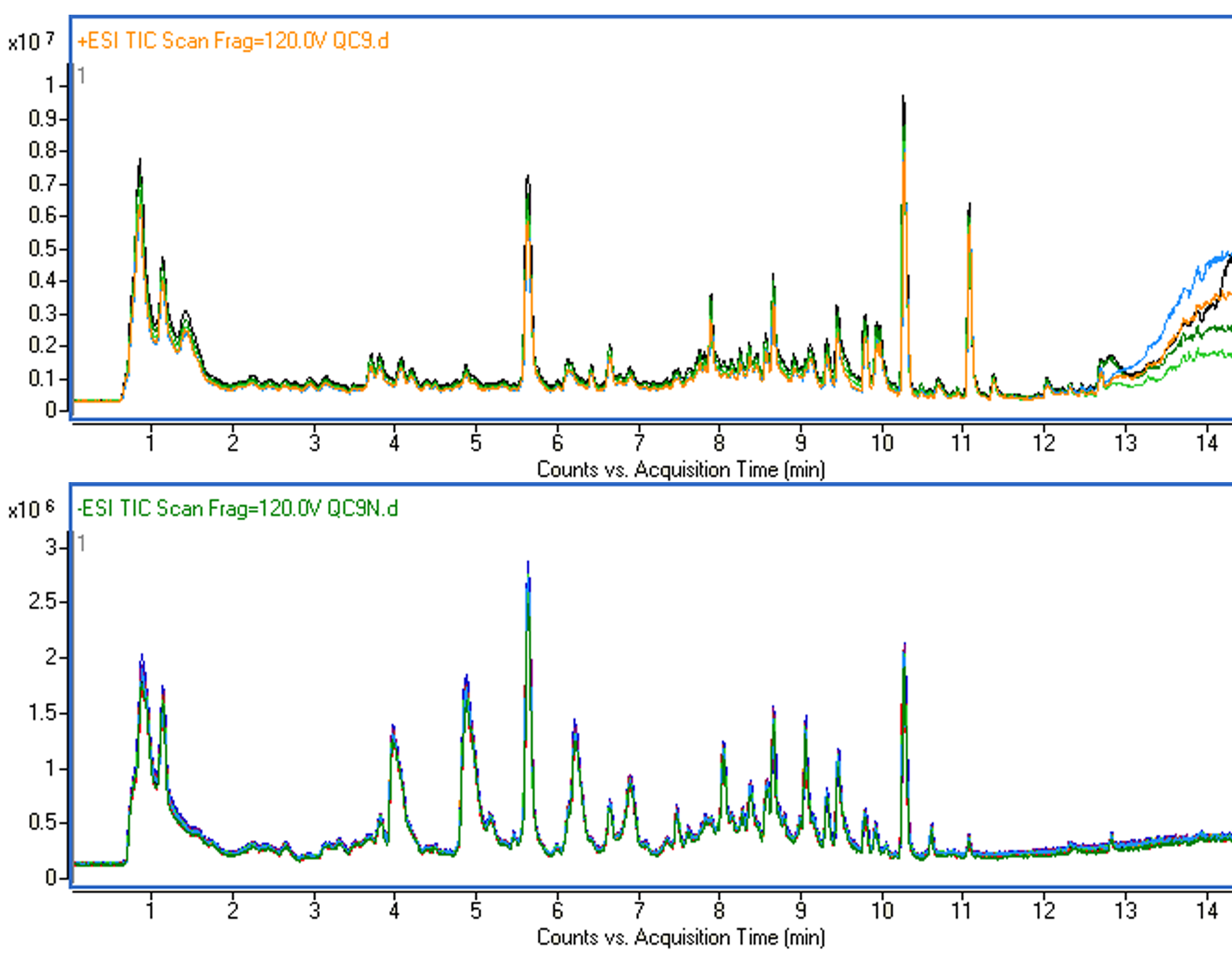

Supplement: Supplementary file 1 [file molecules-24-00253-s001.zip › Supplementary Files/Fig. S2 Quality control superposed graph.tif]
